# Comparison of ring artifact removal methods using flat panel detector based CT images

**DOI:** 10.1186/1475-925X-10-72

**Published:** 2011-08-17

**Authors:** Emran M Abu Anas, Jae G Kim, Soo Y Lee, Md K Hasan

**Affiliations:** 1 Department of Electrical and Electronic Engineering, Bangladesh University of Engineering and Technology, Bangladesh; 2 Department of Biomedical Engineering, Kyung Hee University, Kyungki 446-701, South Korea

## Abstract

**Background:**

Ring artifacts are the concentric rings superimposed on the tomographic images often caused by the defective and insufficient calibrated detector elements as well as by the damaged scintillator crystals of the flat panel detector. It may be also generated by objects attenuating X-rays very differently in different projection direction. Ring artifact reduction techniques so far reported in the literature can be broadly classified into two groups. One category of the approaches is based on the sinogram processing also known as the pre-processing techniques and the other category of techniques perform processing on the 2-D reconstructed images, recognized as the post-processing techniques in the literature. The strength and weakness of these categories of approaches are yet to be explored from a common platform.

**Method:**

In this paper, a comparative study of the two categories of ring artifact reduction techniques basically designed for the multi-slice CT instruments is presented from a common platform. For comparison, two representative algorithms from each of the two categories are selected from the published literature. A very recently reported state-of-the-art sinogram domain ring artifact correction method that classifies the ring artifacts according to their strength and then corrects the artifacts using class adaptive correction schemes is also included in this comparative study. The first sinogram domain correction method uses a wavelet based technique to detect the corrupted pixels and then using a simple linear interpolation technique estimates the responses of the bad pixels. The second sinogram based correction method performs all the filtering operations in the transform domain, i.e., in the wavelet and Fourier domain. On the other hand, the two post-processing based correction techniques actually operate on the polar transform domain of the reconstructed CT images. The first method extracts the ring artifact template vector using a homogeneity test and then corrects the CT images by subtracting the artifact template vector from the uncorrected images. The second post-processing based correction technique performs median and mean filtering on the reconstructed images to produce the corrected images.

**Results:**

The performances of the comparing algorithms have been tested by using both quantitative and perceptual measures. For quantitative analysis, two different numerical performance indices are chosen. On the other hand, different types of artifact patterns, e.g., single/band ring, artifacts from defective and mis-calibrated detector elements, rings in highly structural object and also in hard object, rings from different flat-panel detectors are analyzed to perceptually investigate the strength and weakness of the five methods. An investigation has been also carried out to compare the efficacy of these algorithms in correcting the volume images from a cone beam CT with the parameters determined from one particular slice. Finally, the capability of each correction technique in retaining the image information (e.g., small object at the iso-center) accurately in the corrected CT image has been also tested.

**Conclusions:**

The results show that the performances of the algorithms are limited and none is fully suitable for correcting different types of ring artifacts without introducing processing distortion to the image structure. To achieve the diagnostic quality of the corrected slices a combination of the two approaches (sinogram- and post-processing) can be used. Also the comparing methods are not suitable for correcting the volume images from a cone beam flat-panel detector based CT.

## Background

Ring artifacts are common features in digital X-ray flat panel detector (FPD) based computed tomography imaging. Defective detector elements such as dead pixels in a CCD with non linear responses to the incoming intensity will create sharp rings in the reconstructions of width of one or two pixels. This type of sharp ring is also known as varying intensity rings. Similar artifacts also arise from the imperfect scintillator screens, i.e., the screens affected by scratch, dust or dirt [1]. Mis-calibrated detector pixels, e.g., due to beam instabilities give rise to wider and less marked rings instead [2,3]. It is reported that the ring artifacts can also arise from the monochromator [1], due to thermal processes in CCD [4], changes in temperature or beam strength [5] etc. As the gray value of the reconstructed images are affected by these ring artifacts, it is necessary to cancel them, otherwise, analysis after reconstructions, e.g., noise reduction or segmentation of image information, becomes significantly difficult [6].

Minimization of ring artifacts is possible by using flat-field correction [7], movable detector array [8,9], adequate scanning protocols (e.g., dual gain calibration technique [10]). It is, however, difficult to completely avoid such artifacts and hence to achieve highest quality reconstruction solely by experimental measures [2]. An effective way to eliminate the ring artifacts is the sinogram processing during the reconstruction [2-5,11-21]. Another promising technique to remove these artifacts is the image space processing also known as post processing [6,22-24]. In [24] a method is developed to correct the ring artifacts using a priori information of the attenuation coefficients in some areas of a CT slice. An algorithm is proposed in [4] that is based on the theory of inverse and ill-posed problems. The main idea to correct the ring artifacts is to minimize the Tikhonov's functional. A sinogram based ring removal method is proposed in [14] that is shown to be effective for correcting the 'regular' (the strength of ring artifact does not depend on the rotation angle) ring artifact structure. On the other hand, anisotropically attenuating objects (e.g., object has a large aspect ratio) and defective detector elements are responsible for 'irregular' ring artifact. The key concern of [25] is to suppress this type of ring artifacts.

It is, however, yet unknown which categories of algorithms is more effective in removing ring artifacts as no comprehensive performance analysis of the two categories of algorithms has been made from a common platform considering diverse complexity of the ring artifact problem. Furthermore, the ability of these algorithms in removing the ring artifacts from 3-D cone beam volume CT (CBVCT) images is also investigated. Moreover, different CT instruments (e.g., micro- and dental-CT) with different pixel size, detector area and, different tube current are also used to examine the performance of these methods. To the best of authors' knowledge, there have been no reports on the comparison of ring artifact removals from both micro-CT and dental-CT images. Finally, quantitative comparison is also provided to numerically evaluate the strength of the comparing methods in correcting the ring artifacts.

In this work, an extensive comparative study between the sinogram processing and post processing techniques is done from a common platform to reveal the strength and weakness of the representative algorithms selected from the two aforesaid categories.

## Methods

A brief discussion on the reported ring elimination techniques whose performances are to be evaluated and compared is presented in this section. As the ring artifacts in the reconstructed tomographic image are due to the stripe artifacts in the sinogram, therefore, the sinogram based methods actually remove stripe artifacts from the sinogram image and then use the filtered back projection (fan or parallel beam reconstruction) algorithm to convert the corrected sinogram into a 2-D ring-free fan or parallel beam CT image. In multi-slice CT instruments, ring correction operation is performed on the sinogram image whereas in cone beam CT, an algorithm can be designed to work on the projection image (responses of the 2-D FPD for a particular view/angle). Unlike the fan or parallel beam based multi-slice CT, FDK algorithm [26] is used for the reconstruction in the cone beam geometry based CT. However, sinogram processing based techniques are often adapted to cone beam geometry based projection images by first constructing sinograms from the projections and then transferring back to the projection domain after correction [15-17,19]. The differences between the multi-slice CT (fan or parallel beam CT) and cone beam CT are described in [13]. A sinogram based ring artifact correction technique is presented in [3] that exploits the frequency property of a stripe-corrupted sinogram. Vertical stripes in a sinogram image will appear as high intensity along the horizontal line in Fourier transformed sinogram image. A computationally efficient numerical filter (Butterworth low-pass filter) is then utilized to suppress these horizontal line defects in the frequency domain. As significant image information is also located in the horizontal line of the Fourier transformed sinogram image, this method is not much reliable to remove the stripe artifacts from the sinogram image. An improved version of this method is recently published in [2] and this technique performs Fourier filtering on the coefficients of 2-D wavelet decomposed vertical detail band instead of on the original sinogram image. In [12,5] efficient and fast methods have been proposed to remove the stripes by smoothing the sum curve computed from the corrupted sinogram and then normalizing the sinogram. The design of the smoothing filter, however, differs in the two approaches. Nevertheless, these methods fail to remove the varying intensity sharp rings, because the normalization procedure is inappropriate to eliminate this type of rings [5,15-17]. Most recently, works based on center-weighted median filter [15] and morphological filters [16] and 1-D WMA/VWMA filters [19] have been reported to eliminate the ring artifacts from a tomographic image. But these techniques cannot suppress different types of ring artifacts effectively. Moreover, the algorithm in [16] is relatively computationally expensive.

A polar domain based post-processing algorithm is presented in [6] and it, at first, selects a region of interest (ROI) by separating the object from the image background and, then extracts the artifact template vector. Finally, the artifact template vector is subtracted from each row of the polar image in order to get the corrected polar image. Recently, two post-processing algorithms are reported in [22] and they are based on median and mean filtering of the reconstructed images but each working in different geometric planes, i.e., cartesian coordinate and polar coordinate. It is demonstrated in [22] that the ring artifact can be better removed in polar coordinate than that in the cartesian coordinate.

In this study, two algorithms from each of the two categories, i.e., total four algorithms are chosen to evaluate and compare their performances for various ring patterns generated by the CT imaging system and the objects. The first correction method is based on the sinogram processing and has been derived from [13]. In fact, the algorithm in [13] removes the ring and radiant artifacts from the 3-D CBVCT images obtained using a 2-D flat panel digital detector. As the interest of this study is to compare the performance of the algorithms that can deal with parallel or fan beam projection images of multi-slice CT as well, some modifications are proposed to suit the algorithm in [13] to fan beam or parallel beam CT images. The second correction technique is the wavelet-Fourier method presented in [2] that uses both wavelet and Fourier filtering to remove the stripe artifacts from the sinogram images. The two post-processing algorithms that operate on the reconstructed images are taken from [6] and [22]. As the ring artifact correction in polar coordinates (RCP) is more effective than that in the cartesian coordinates [22], therefore, only the RCP method from [22] is examined in our study. Finally, in this comparative study we also include our recently published sinogram based ring correction method [17]. In the following, the proposed modification of the wavelet based method in [13] and the basic ideas behind the rest of the four algorithms are briefly explained. The limitations of each of the algorithm are also discussed alongside.

### Modified wavelet plus normalization (MWPN) method

**Figure 1:**
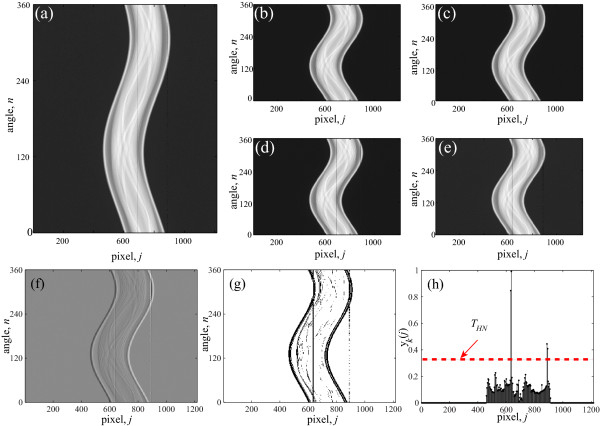
**The correction process shown for the modified wavelet method**. (a) Sinogram image of an electrolytic capacitor, (b-e) subset sinograms, *P_k_*(*n*, *j*) for *k *= 1 to 4, respectively, (f-g) image view of *D_k_*(*n*, *j*) and *T_k_*(*n*, *j*) for *k *= 4, respectively, (h) variation of *y_k_*(*j*) with *j*. A threshold *T_HN _*should be properly selected so that only the bad pixels are detected and the edges remain outside of detection.

In the original work [13], the flat-field image is used to detect the positions of corrupted pixels. Since in this work the other comparing algorithms correct the ring artifacts using the sinogram/CT image only, therefore, in the proposed modified method, all operations are performed on the sinogram image instead of the flat-field image to ensure fair comparison. The first step is to decompose the original, uncorrected sinogram *P*(*n*, *j*) (shown in Figure 1(a)) into a set of subsets. Here, 1 ≤ *n *≤ *n_v _*and 1 ≤ *j *≤ *j_d _*and, *n_v _*is the total number of projections (views) taken and *j_d _*is the total number of the detector pixel elements. The purpose of decomposing the sinogram is to separate the band of stripes from each other. If the decomposition level is set to *L*, then the subset sinograms can be written as:

(1) $$\begin{array}{c} P_{k}\left(n,j\right)\;=\;P\left(n,j\right):\\ \;for\;1\leq j\leq j_{d}\;\;and\;mod\left(j,L\right)=mod\left(k,L\right) \end{array}$$

where, 1 ≤ *k *≤ *L*. mod(*j*, *L*) is the remainder left after dividing *j *by *L*. Using *L *= 4, the original sinogram is decomposed into four subset sinograms as shown in Figures 1(b-e). Now, 1-D Haar wavelet decomposition is performed over each row of the subset sinogram *P_k_*(*n*, *j*) and as a result, a new matrix *D_k_*(*n*, *j*) is obtained that contains the fine scale component. Therefore, the relationship between *D_k_*(*n*, *j*) and *P_k_*(*n*, *j*) can be written as

(2) $$\begin{array}{c} D_{k}\left(n,j\right)\;=\;\frac{-1}{\sqrt{2}}\left(P_{k}\left(n,\;j\right)-P_{k}\left(n,\;j-L\right)\right):\\ \;for\;1\leq j\leq j_{d}\;\;and\;\text{mod}\left(j,\;L\right)=\text{mod}\left(k,\;L\right) \end{array}$$

where, mod(*j*, *L*) is the remainder left after dividing the *j *by *L*. Generally in the 1-D wavelet operation, a downsample operation is performed after filtering (e.g., in this case equation (2)) the original signal. In this work, this downsampling operation on the filtered signal is not performed as it may exclude any bad pixels to be detected. The gray-scale plot of *D_k_*(*n*, *j*) for *k *= 4 is shown in Figure 1(f). Now, to detect the discontinuous points in *D_k_*(*n*, *j*) as suggested by the original work [13], a test is performed. If *D_k_*(*n*, *j*) ≤ *m*_1 _- *w*_0_*m*_2 _or *D_k_*(*n*, *j*) ≥ *m*_1 _+ *w*_1_*m*_2_, then, the point (*n*, *j*) (where, 1 ≤ *j *≤ *j_d _*and mod(*j*, *L *) = mod(*k*, *L*)) is said to be discontinuous, otherwise, it is continuous. Here, $w_{0}=k_{0}m_{g}\sqrt[{4}]{m_{g}}$, $w_{1}=k_{0}\left(0.9-m_{g}\right)\sqrt[{4}]{m_{g}}$, *m*_1 _= Mean(*D_k_*(*n*, *j*)), *m*_2 _= SD(*D_k_*(*n*, *j*)), $m_{g}=\text{Mean}\left(P_{k}^{n}\left(n,j\right)\right)$ and, *k*_0 _is an experimentally determined constant and $P_{k}^{n}\left(n,\;j\right)$ is the normalized version of *P_k_*(*n*, *j*), i.e., $P_{k}^{n}\left(n,\;j\right)=\frac{P_{k}\left(n,j\right)-P_{min}}{P_{max}-P_{min}}$. Here, the minimum value of *P_k_*(*n*, *j*) is *P_min _*and the maximum value of *P_k_*(*n*, *j*) is *P_max_*. SD(·) denotes the standard deviation operation. Now, a binary template matrix *T_k_*(*n*, *j*) of the same size as *D_k_*(*n*, *j*) is created, which contains either 1 or 0, i.e., if (*n*, *j*) contains discontinuity then, *T_k_*(*n*, *j*) = 0, otherwise it is 1. The distribution of the discontinuous points is shown in Figure 1(g). It is observed from this distribution that along the projections of the bad pixels the points are more discontinuous than those of the good pixels. As the target is to detect the bad pixel locations, the sum *y_k_*(*j*) of the gray values of each pixel *j *of the logically inverted matrix of *T_k_*(*n*, *j*) is calculated over each projection: $y_{k}\left(j\right)=\frac{\sum_{n}{\bar{T}}_{k}\left(n,j\right)}{n_{v}}$, where, ${\bar{T}}_{k}\left(n,\;j\right)$ is the logically inverted matrix of *T_k_*(*n*, *j*). In the numerator *n_v _*is used to eliminate any effect of scaling on *y_k_*(*j*). The variation of *y_k_*(*j*) over *j *is shown in Figure 1(h). Now, a pixel *j *is detected as a bad pixel if *y_k_*(*j*) ≥ *T_HN_*, where, *T_HN _*is an experimentally determined threshold. The main drawback of this method is its failure of detecting the positions of the weak stripes. It is not possible to lower the value of *T_HN _*in order to detect the weak stripes, because doing this would falsely detect the edges of the sinogram image. Another problem of this technique is that due to the wavelet operation (please see equation 2), a single discontinuity at (*n*, *j*) = (*n*_0_, *j*_0_) in *P_k_*(*n*, *j*) leads to two high magnitudes in *D_k_*(*n*, *j*) at (*n*, *j*) = (*n*_0_, *j*_0_) and (*n*, *j*) = (*n*_0_, *j*_0 _+ *L*) and therefore, if a stripe is located at the *j *= *j*_0_-th pixel, then both the pixels at *j *= *j*_0 _and *j *= *j*_0 _+ *L *are detected as bad pixels because of *y_k_*(*j*_0_) ≥ *T_HN _*and *y_k_*(*j*_0 _+ *L*) ≥ *T_HN_*.

The operations of the algorithm on a particular subset sinogram (for *k *= 4) is shown in Figure 1. To detect the positions of all bad pixels, *k *must be varied from 1 to *L *(e.g., 4 in this case). To avoid the case of the stripes which were separated in the original sinogram, but form band stripes in the subset sinogram, the decomposition level (*L*) is varied from 1 to *L_max _*in order to detect all (isolated or band) types of stripes, where, *L_max _*is the maximum number of the decomposition levels. After detecting the positions of all stripe creating pixels, the correction is done by using the linear interpolation technique in the positions of the detected stripes only. The corrected sinogram thus obtained is denoted by *P'*(*n*, *j*).

It is observed from the correction steps shown in Figure 1 that this method cannot detect the weak stripes as it selects the threshold so as to avoid the detection of any image edge elements as discontinuous points. Therefore, to correct the weak stripes the moving average filter method presented in [12] can be used. To do this, first the sum curve, *y*'(*j*) is calculated from the partially corrected sinogram *P*'(*n*, *j*): *y*'(*j*) = Σ*_n _P*'(*n*, *j*). Then the sinogram is scaled by the factor *y_s_*(*j*)*/y*'(*j*), i.e., $\hat{P}\left(n,\;j\right)=P^{\prime }\left(n,j\right)\frac{y_{s}\left(j\right)}{y^{\prime }\left(j\right)}$, where $\hat{P}\left(i,\;j\right)$ is the corrected sinogram and *y_s_*(*j*) is the smoothed version of *y*'(*j*) applying a non-causal moving average filter. The difference equation of this filtering scheme looks like

(3) $$y_{s}\left(j\right)=\frac{1}{2N+1}\overset{N}{\underset{k=-N}{\sum }}y^{\prime }\left(j+k\right)$$

where, *N *is the span factor.

### Wavelet-Fourier (WF) method

**Figure 2:**
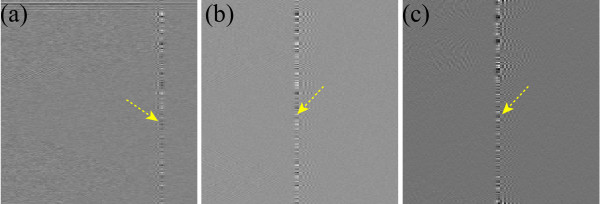
**Drawbacks of the wavelet-Fourier method**. (a-c) Magnified image view of the coefficients of the horizontal, diagonal and filtered (Fourier) vertical detail bands, respectively. These three bands' coefficients must be free from stripe information. But we see that vertical stripes are present in these three detail band coefficients as marked by arrows.

First, 2-D multi-resolution wavelet decomposition is performed in [2] on the corrupted sinogram *P *(*n*, *j*) in order to exclusively condense the information of vertical stripes to the coefficients of vertical detail band and to the low frequency band. Next, 2-D wavelet decomposition is performed on the low frequency band repeatedly, until the finally remaining low frequency band is free from the stripe information. Hence, the highest decomposition level (*L*) required is dependent on the maximum stripe width. For a sufficiently large *L*, the impact of stripe information on the low pass coefficients becomes negligible. Fourier filtering is now performed only over the coefficients of the vertical detail band to remove the stripe information. The full MATLAB code of this algorithm is provided in [2]. This method works well when a sinogram is corrupted by ideal stripes [2] (pixel responses' are constant) and stripes generated from the mis-calibrated detector elements. But if the sinogram is corrupted by the varying intensity rings, then the performance of this method is found to be not satisfactory. In this case, the coefficients of the horizontal and diagonal detail bands also contain stripe information and the suggested Fourier filtering is not much effective to eradicate the stripe information from the coefficients of the vertical detail band. Figures 2(a-c) show the coefficients of the horizontal, diagonal and filtered vertical detail bands (zoomed view), respectively, after the first level 2-D wavelet decomposition of the sinogram image shown in Figure 1(a). It indicates that the vertical stripe information exists in these detail band coefficients. And as the corrected sinogram is constructed using these stripe corrupted detail band coefficients, it will contain vertical stripe information and the diagnostic quality of reconstructions will be degraded.

### Ring corrections using homogeneity test (RCHT) (Sijbers 2004) method

At first, a thresholding is performed on the reconstructed image (**I**) with a view to separate the object from the background [6]. It is not crucial to select an accurate threshold (*T_ROI_*) as the effect of an improperly chosen threshold will be compensated by the subsequent morphological operations (dilation + masked erosion + erosion + masked dilation). A binary image (**B**) is thereby generated that serves as a ROI to suppress the ring artifacts.

Within the ROI, ring artifacts in the reconstructed image are corrected. In summary, the steps involving the ring artifact correction are:

1. At first, the reconstructed CT image is transformed into polar coordinates.

2. A sliding window (column size *W*) is selected and a set of homogeneous rows that meet the homogeneity criterion (signal variance *<*threshold *T*) are detected and, an artifact template vector is then generated.

3. The line artifacts are corrected in the polar transformed image based on the set of artifact templates.

4. Finally, the polar image is transformed back into the cartesian coordinates to get the corrected 2-D reconstructed image.

It is to be noted that these operations are performed only on the pixels belonging to the ROI. In this method, the intensity of a ring is assumed to be constant throughout a ring structure because the same artifact template vector is subtracted from each row for suppressing the line artifact. This simple assumption, however, is not true for the often seen varying intensity rings and the performance of this algorithm is, therefore, expected to be not satisfactory for the removal of varying intensity ring artifacts.

### Ring correction in polar coordinate (RCP)

The details of this ring correction method are presented in [22]. In short, the steps of this algorithm in polar coordinate are briefly outlined below:

1. Two thresholds (lower threshold *T_min _*and upper threshold *T_max_*) are used so that no new artifacts are generated.

2. Median filtering is performed on the thresholded image in the radial direction.

3. To identify the ring artifact structures, the difference between the median filtered image and the thresholded image is computed. A second threshold (*T_RA_*) is then used to ensure that the ring artifact structures pass the filtering while the bone structures are excluded.

4. Low-pass (mean) filtering is performed in the azimuthal direction in order to provide a difference image which contains only the artifact structures.

Finally, inverse transformation of the artifact image into cartesian coordinates yields the ring structures in this coordinate system. Then this artifact image is simply subtracted from the initial image to get the corrected image. The authors have shown that the method can remove ring artifacts from the C-Arm CT and micro-CT images [22,23].

If varying intensity rings are present in a reconstructed CT image, then the difference image generated after step (3) expectedly contains the varying intensity rings. But the subsequent mean filtering of the difference image in the azimuthal direction certainly fails to hold the correct varying intensity ring structures in the difference image, because the varying intensity rings generally contain significant high frequency information and the mean filtering removes this information from the difference image. As the difference image does not always contain the appropriate ring structures, thus, the acceptable quality of the corrected image may not be achieved as it is obtained after subtracting the difference image from the original image.

### Strength based ring correction(SBRC) method

In this method [17], at first the sinogram is windowed to create a sub-sinogram by keeping the pixel of examination at the center position in the sub-sinogram. The other pixels in the sub-sinogram are selected from a polyphase component of the sinogram. The polyphase concept is exploited to detect the band stripes correctly. The maximum number of polyphase levels (*l_m_*) is equal to the maximum width of the band stripes in the sinogram. As stripes create discontinuity in the sinogram, a first-derivative-based algorithm is adopted to detect the stripes from a sub-sinogram. Exploiting the fact that the stripes generating from the defective detector elements are much stronger than those from the mis-calibrated detector elements, a derivative based mathematical index is calculated to measure the strength of the stripes. Finally, the strong and weak stripes are differentiated by comparing the index with two appropriately selected thresholds (e.g., *r_min _*and *r_max_*). Two dimensional (2D) variable window moving average (2D-VWMA) and weighted moving average (2D-WMA) filters are used in a combined way to suppress the strong stripes because they require total reconstruction from the neighbors. On the other hand, the normalization method is used to eliminate the weak stripe artifacts from the sinogram. As this method classifies the ring artifacts into two groups and corrects each group by appropriate schemes, therefore, this method successfully suppresses different types of ring artifacts from the CT images.

### Description of the data sources

The test images were acquired with a home made micro-CT and a dental-CT. The micro-CT consists of a CMOS FPD and a micro focus X-ray tube (L8121-01, Hamamatsu, Japan). For micro-CT experiments, two FPDs (C7943CA-02, C7942CA-02, Hamamatsu, Japan) were used. The FPDs consist of 1216 × 1220 and 2240 × 2240 effective matrix of transistors, and photodiodes with a pixel pitch of 100*μ*m and 50*μ*m, respectively, and a CsI:Tl scintillator. In one micro-CT experiment (bone image), we have used the detector (C7942CA-02) in 2 × 2 binning mode so that the active matrix size becomes 1120 × 1156. The CMOS FPD (Ray, Korea) in the dental-CT consists of 4096 × 1024 matrix of transistors, and photodiodes with a pixel pitch of 48*μ*m and a CsI:Tl scintillator. Here also we have used the FPD in 2 × 2 binning mode and, therefore, the active matrix size has become 2048 × 512. Both the micro- and dental-CT machines are based on cone beam geometry. Unfortunately, our CT system is not calibrated in Hounsfield unit (HU). Therefore, all the uncorrected CT images are first normalized in order to make the maximum pixel intensity 1.0 (arbitrary unit). Then we scale the corrected images by applying the corresponding normalization factor of the uncorrected images [18].

## Results and Discussion

In this section, we test the performance of all the algorithms using some selective real CT images. All the methods use some parameters which need to be adjusted from image to image to achieve good results. For any method, parameter selection is an important point for effective removal of the ring artifacts. The first method (MWPN [12,13]) uses four parameters: maximum number of decomposition levels (*L_max_*), discontinuity index (*k*_0_), threshold (*T_HN_*) and span factor (*N*). *L_max _*is chosen in such a way that every stripe in the initial image gets isolated in any one of the subset sinograms. Generally, it is made equal to the maximum width of the band stripes. But even if a low value of *L_max _*(e.g., 1 or 2) is selected, it may work too. Because in the first stage (up to applying the normalization [12]), the aim is to remove the strong or varying intensity rings and they generally appear in at most two pixel width. The value of *k*_0 _has an impact on the detection of the discontinuous points. A low value of *k*_0 _leads to more points to be decided as discontinuous. The threshold *T_HN _*should be carefully selected so that it detects all the bad pixels but excludes the edge positions. Finally, the span factor (*N*) should be appropriately selected to eliminate the weak rings. The WF [2] has three parameters: decomposition level (*L*), mother wavelet and damping coefficient (*σ*). Here, the value of *L *is equal to the maximum stripe width. There are various choices available to select the mother wavelet, e.g., db1, db2, db3, db25, db31, db41, db42, db43 etc. Selection of a smaller length wavelet results in a low computation time, but at the cost of poor image quality. On the contrary, choosing longer length wavelets (e.g., db41, db42, db43) results in the best quality reconstructed image, but with higher computational burden. We prefer the second option to ensure good diagnostic quality of the reconstructed images. A low value of the damping coefficient (*σ*) is insufficient to eliminate the stripe information whereas a high value leads to a blurring effect in the tomographic images. The third method called the ring corrections using sliding window (RCHT) [6] has three parameters needed to be adjusted to obtain ring-free slices. They are threshold in ROI selection (*T_ROI_*), column size (*W*) and homogeneity threshold (*T*). The selection of the first parameter is not critical as mentioned earlier. *W *should be chosen within the range [100-150] [6]. On the other hand, the value of *T *is dependent on the ring artifacts. The less pronounced the line artifacts, the smaller the value of *T *can be chosen.

In case of the RCP [22] method, the filter width is selected as suggested in the original work, e.g., radial median filter width in polar coordinates, $M_{Rad}^{P}=15$; azimuthal filtering in polar coordinates, $M_{AZi}^{P}=40$. On the other hand, the distance between the support points in the azimuthal direction $\left(d_{AZ}^{P}\right)$ for the polar coordinate is needed to be adjusted for our test CT images. We set $d_{AZ}^{P}$ equal to 0.7°, instead of 0.8°. In the original work [22], the distance between the support points in the radial direction (*d_RA_*) for both the cartesian and the polar coordinates is determined from the scanner geometry. In our case, this parameter is set to 1.0 for the polar coordinate $\left(d_{RA}^{P}\right)$. The RCP method uses three thresholds (*T_min_*, *T_max _*and *T_RA_*) for image segmentation and bone structure elimination. These three thresholds are considered in HU unit in the original work. Since our CT images are not adjusted in HU unit, therefore, these three thresholds are selected as mentioned in our previous work [18]. In the following at first we present the comparative results of the MWPN, WF, RCHT and RCP methods and then in a separate section we show the results of our SBRC method.

### Removal of varying intensity rings in a structural object

**Figure 3:**
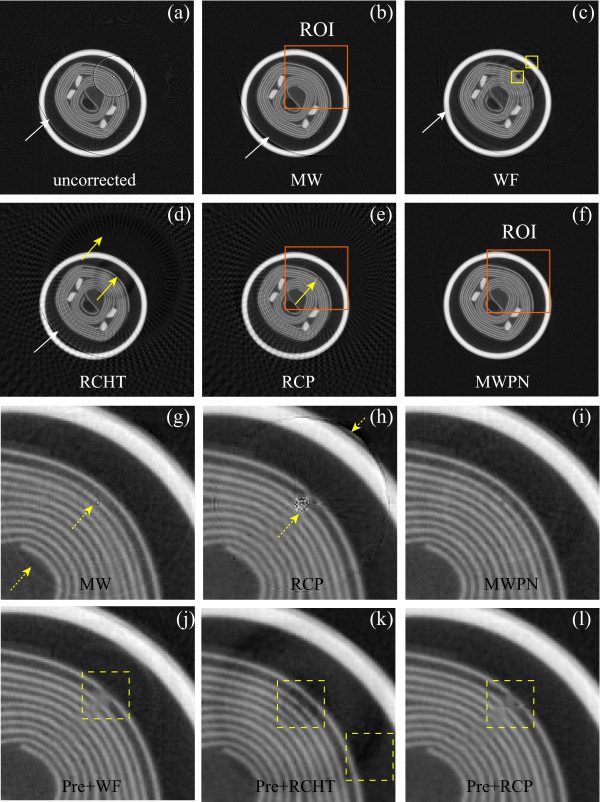
**Removal of ring artifacts from an electrolytic capacitor image**. (a) Uncorrected, original image. (b-f) Corrected images by applying the modified wavelet method without normalization (*L_max _*= 3, *k*_0 _= 5.85 and *T_HN _*= 0.33), WF (*L *= 3, wname='db43' and *σ *= 7.5), RCHT (*W *= 125 and *T *= 0.0023), RCP and MWPN (*L_max _*= 2, *k*_0 _= 5.85 and *T_HN _*= 0.33, *N *= 4) methods, respectively. Without using the normalization technique, the modified wavelet method alone is not appropriate to delete the weak rings (such a ring is shown in (b) as marked by an arrow). Wavelet-Fourier method blurs the image in different regions as marked by boxes. (g-i) Zoomed view of the ROIs (b), (e) and (f), respectively. The effectiveness of the normalization technique in removing the weak rings is evident in (f). (j-l) Effect of the pre-correction using [17]. Corrected ROIs are shown in (j-l) by the WF (*L *= 3, wname='db43' and *σ *= 7.5), RCHT (*W *= 125 and *T *= 0.0023) and RCP methods (applied on the pre-corrected image), respectively. It is illustrated that as the varying intensity rings are pre-corrected, therefore, the remaining mis-calibration rings are suppressed by these three methods. But blurring is observed at the ring location marked by dashed boxes due to the longer filter lengths in these three methods. Same window settings 'C/W = 0.5880/0.1686' are used for all sub-figures.

We choose a ring artifact corrupted micro-CT slice of an electrolytic capacitor image shown in Figure 3(a). It is very important to clearly visualize the inside micro-structure of such a highly structural fabrication product because any misinterpretation of the structure will severely affect the fabrication process (e.g., new design might be sought, though the previous design was perfect). From the uncorrected image (Figure 3(a)) it is very difficult to understand the inside micro-structure clearly. Hence, the correction methods should be smart enough to achieve the required quality. The corrected images by using the modified wavelet without normalization, WF, RCHT, RCP and MWPN methods are presented in Figures 3(b-f). Figures 3(b-f) indicate that the MWPN method performs the best amongst these methods. The WF method blurs the image at different locations marked by boxes in Figure 3(c) while, the RCHT and RCP methods fail to remove the artifact completely (marked by arrows in Figures 3(d-e)). But the RCP method deletes the ring marked by arrow in Figure 3(e) whereas this ring is remained in the other corrected Figures 3(b-d). To demonstrate the effect of the normalization technique on the corrected image, a region of interest (ROI) is chosen around the center of rotation as marked in Figures 3(b), (e-f). Now we zoom the three ROIs as shown in Figures 3(g-i), respectively. It is visible from the zoomed ROIs (Figures 3(g-h)) that some rings still remain in the corrected image around the center of rotation when the RCP or only the modified wavelet method is used. If the normalization method of correction is included in the modified wavelet, then these weak rings can be deleted as shown in Figure 3(i). As mentioned before, WF, RCHT and RCP methods are particularly weak in erasing the sharp varying intensity ring artifacts. The results presented in Figures 3(c-e) also support this observation. Therefore, to test these methods without such artifact a pre-correction of all the varying intensity ring artifacts is performed first on the uncorrected capacitor image by using the SBRC algorithm presented in [17]. In order to apply the SBRC method as a pre-correction technique, at first the stripe strength measuring index is calculated for all the pixels. Then the defective pixels are recognized by comparing the calculated mathematical index with *r_max_*. If the index value for a particular pixel is greater than *r_max_*, then this pixel is detected as defective pixels and 2-D VWMA/WMA filters are used to correct the responses of these detected pixels. Here, *r_max _*= 15 and *l_m _*= 5 are used in the SBRC method as a pre-correction technique. Note that the values of these two parameters are kept constant throughout all the experiments in this paper when the SBRC method is used as a pre-correction technique. Now, the WF, RCHT and RCP methods are applied on the pre-corrected image. Figures 3(j-l) display the corrected ROIs by these three methods, respectively. As the sharp rings are removed prior to the application of the three methods, the performance of all these methods is now better than the previous case in suppressing the ring artifacts. But, blurring effect is observed around the region of the ring location. As the ring artifact is located at the structural region and the three methods, especially the WF and RCHT methods use longer length filtering operation, the region of ring location is blurred as marked by the dotted boxes in Figures 3(j-l).

### Ring artifact removal from micro-CT and dental-CT images

**Figure 4:**
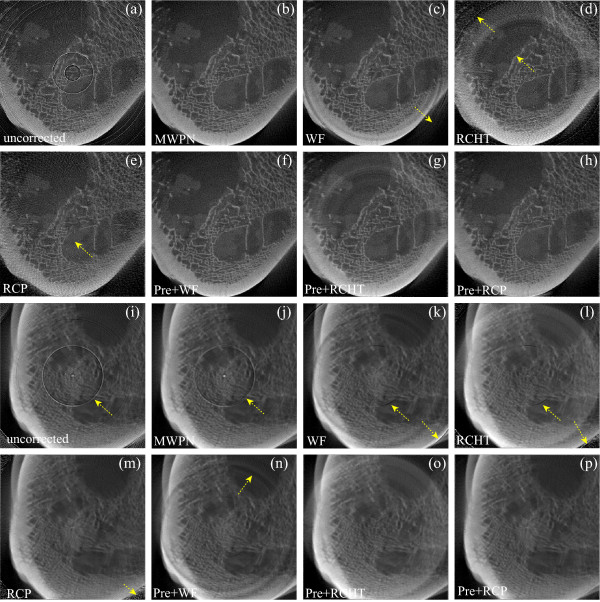
**Removal of ring artifacts from the micro-CT and dental-CT images of an animal bone**. (a) Uncorrected bone image from micro-CT machine, (b-e) corrected images by using the MWPN (*L_max _*= 1, *k*_0 _= 5.10, *T_HN _*= 0.10 and *N *= 2), WF (*L *= 4, wname='db42' and *σ *= 10.0), RCHT (*W *= 125 and *T *= 0.0025), and RCP methods, respectively. (f-h) Effect of pre-correction on the WF, RCHT and RCP methods. Corrected images by using the WF (*L *= 2, wname='db41' and *σ *= 0.5), RCHT (*W *= 125 and *T *= 0.0005), and RCP methods applying on the pre-corrected image are displayed in (f-h), respectively. (i) Uncorrected bone image from dental-CT machine, (j-m) corrected images by using the MWPN (*L_max _*= 5, *k*_0 _= 5.10, *T_HN _*= 0.10 and *N *= 8), WF (*L *= 5, wname='db42' and *σ *= 10.5), RCHT (*W *= 125 and *T *= 0.0023), and RCP methods, respectively. (n-p) Applying correction on the pre-corrected image by using the WF (*L *= 5, wname='db42' and *σ *= 3.5), RCHT (*W *= 125 and *T *= 0.0019), and RCP methods, respectively. The window settings for (a-h) are 'C/W = 0.4132/0.2994' and that for (i-p) are 'C/W = 0.4714/0.1998'.

For extensive comparison of ring artifact removal methods in different CT imaging conditions and detector types, now, we use raw projection data acquired from a micro-CT and a dental-CT. In both scanning, we have used the same magnification ratio of 1.4 so that we always have the same size images from the two CTs. We set the tube voltages of the two CTs to the same level, that is, 80 kVp. Tube current of the dental-CT was set to 10mA and that of the micro-CT to 37 *μ*A. The number of views was set to 300 for both the CTs. A hard bone structure is considered here for this experiment. Figures 4(a) and Figure 4(i) show the uncorrected bone CT images obtained from the micro- and dental-CTs, respectively. The corrected images by the MWPN, WF, RCHT and RCP methods are demonstrated in Figures 4(b-e) (for micro-CT) and Figures 4(j-m) (for dental-CT), respectively. It is observed from the uncorrected images (Figures 4(a) and 4(f)) that the ring structures are different in these images as different types of CTs are used to acquire the CT images. From the corrected images (Figures 4(b-e) and Figures 4(j-m)) it is noticed that the WF, RCHT and RCP methods fail expectedly to remove the varying intensity rings. The WF method clears the radiant artifact but this artifact is clearly visible in the corrected images by the RCHT and RCP methods. Now, a pre-correction of the varying intensity rings is done using [17] and the corrected images by the WF, RCHT and RCP methods are demonstrated in Figures 4(f-h) (for micro-CT) and Figures 4(n-p) (for dental-CT), respectively. As the pre-correction is performed, the performance of these methods is quite satisfactory for the micro-CT image as noticed from Figures 4(f-h). On the other hand, for dental-CT a strong band ring marked by a yellow arrow is noticed in Figure 4(i) and this band ring creates consecutive five stripes in the uncorrected sinogram image. Only the RCP method (Figures 4(m), (p)) successfully clears the band ring from the uncorrected bone CT image obtained from the dental-CT. It is observed from Figure 4(i) that the band ring is strong enough, therefore, the MWPN method detects this band structure using the wavelet operation in the first stage. Though the width of the band stripes in the uncorrected sinogram is five pixels, the MWPN method detects consecutive ten pixels as bad pixels for the reason stated earlier. Therefore, the MWPN method uses distant neighborhood information for correcting the ring structure and thereby, fails to delete the band structure adequately. The incapability of the WF method in erasing a wide band ring structure and also the underlying reasons are addressed in [17]. Note that the band ring has disappeared in Figures 4(n-o) due to the pre-correction of ring artifacts, but not because of the effectiveness of the WF and RCHT methods.

### Ring artifact removal from multi-slice images

**Figure 5:**
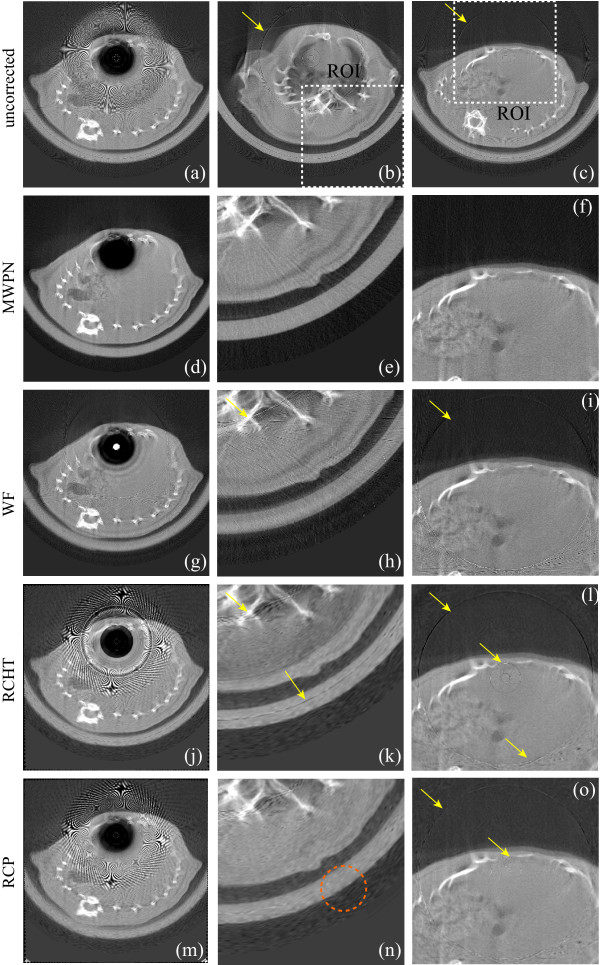
**Removal of ring artifacts from three 2-D slices of the reconstructed rat abdomen image of a 3-D CBVCT image**. (a-c) Uncorrected images, corrected images by using the (d-f) MWPN method (*L_max _*= 1, *k*_0 _= 6.4, *T_HN _*= 0.22 and *N *= 3), (g-i) WF method (*L *= 4, wname='db42' and *σ *= 4.5), (j-l) RCHT method (*W *= 125 and *T *= 0.0025) and (m-o) RCP method. Same window settings 'C/W = 0.2628/0.0481' are used for all sub-figures.

The WF, RCHT and RCP methods are basically designed for the fan or parallel beam geometry based CTs. However, in the cone beam geometry based CTs these methods can be also applied as a 3-D cone beam volume CT (CBVCT) image can be regarded as a collection of 2-D CT slices. In contrast to fan or parallel beam geometry based CT, it is suitable to correct the ring artifacts in CBVCT using a single parameters setting since there are many slices in a 3-D CBVCT image. It would be impractical to manually choose the optimal parameters for each slice. Thus, in this work a single parameter setting is maintained to correct all the slices of the CBVCT image and three of the corrected slices will be presented here. Figures 5(a-c) demonstrate such three slices of a rat abdomen. It is noticed that these three slices are originally little blurry due to the respiratory motion of the live rat. It is observed from these three reconstructed images that the first slice (Figure 5(a)) is severely corrupted by the ring and radiant artifacts. The degree of corruption is not so high in the next two slices (Figures 5(b-c)). Two ROIs are chosen from Figures 5(b-c): i.e., one contains region far from the center of rotation and the other contains region around the center of rotation. Now, a unique parameter setting is maintained for all these three images and the corrected images by using the MWPN, WF, RCHT and RCP methods are presented in Figures 5(d-o) (MWPN: Figures 5(d-f), WF: Figures 5(g-i), RCHT: Figures 5(j-l), RCP: Figures 5(m-o)), respectively. It is to be noted that the rightmost two images for each correction method are the corrected zoomed ROIs (ROIs are shown in Figures 5(b-c)). It is illustrated that none of the comparing methods eliminate the severe ring artifacts from the first slice. In fact, it is not possible for any multi-slice (fan and parallel beam geometry) based ring correction algorithm to completely remove such ring artifacts from 2-D slices. In [23], it is claimed that the 2-D correction algorithm can be used to completely eliminate the ring artifacts from a 3-D image. But observing the first slice it is evident that this claim is partially true. If the next two slices (Figures 5(b-c)) are considered, then it is noticed that only the MWPN method clears all the rings successfully from the two CT slices. As in this analysis varying intensity rings are not pre-corrected, the quality of the images corrected by the WF, RCHT and RCP methods are not satisfactory. The WF technique partially removes the ring artifacts as shown in Figures 5(h-i) (marked by the yellow arrows). The RCP method deletes the rings more than the RCHT method does, but severe blurring is observed around the boundary region of the two layers in the corrected images as shown in Figures 5(n) (marked by circle).

### Removal of ring artifacts at the edges of high contrast object

**Figure 6:**
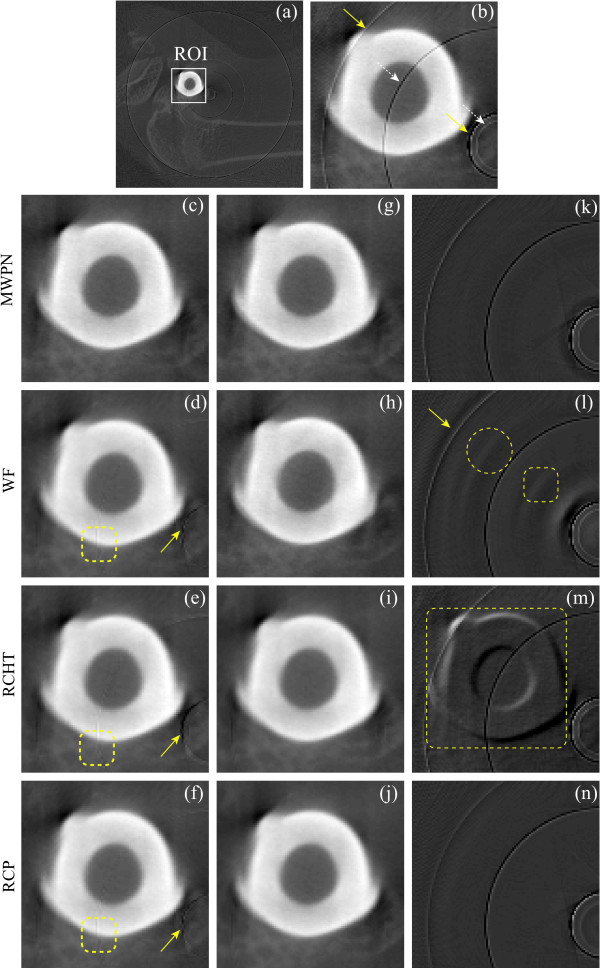
**Removal of ring artifacts from a rabbit bone with a metal implant image**. (a) Uncorrected, initial image, (b) magnified view of the ROI in (a). (c-f) Corrected ROIs by using the MWPN (*L_max _*= 1, *k*_0 _= 8.1, *T_HN _*= 0.25 and *N *= 8), WF (*L *= 4, wname='db42' and *σ *= 8.0), RCHT (*W *= 125 and *T *= 0.0019), and RCP methods, respectively. (g-j) Effect of pre-correction on the MWPN, WF, RCHT and RCP methods. Corrected images by using the WF (*L *= 4, wname='db42' and *σ *= 4.5), RCHT (*W *= 125 and *T *= 0.0021), and RCP methods applying on the pre-corrected image are displayed in (g-j), respectively. (k-n) Difference ROIs between the uncorrected and corrected ROIs (g-j), respectively. Same window settings 'C/W = 0.6716/0.5827' are used for all sub-figures.

Till now, the correction methods have been examined in eliminating the ring and radiant artifacts from the reconstructed images. Now, the effect of the ring correction methods on the object information is investigated. To do so, a reconstructed image is chosen where a high contrast metallic ring is placed in a low contrast rabbit femur (Figure 6(a)). Now, a ROI is chosen as shown in Figure 6(a) and the magnified view of this ROI is demonstrated in Figure 6(b). Such a ROI is always interesting in a ring correction analysis because two rings (marked by yellow arrows) are located at the edge of the high and low contrast medium. The corrected images by using the MWPN, WF, RCHT and RCP methods are presented in Figures 6(c-f), respectively. It is noticed from the corrected images (marked by arrow and box in Figures 6(d-f)) that the performance of all but the MWPN method is not acceptable because of the presence of varying intensity rings. Now, a pre-correction of the varying intensity rings on the uncorrected image is done using [17] and the corrected images by using the MWPN, WF, RCHT and RCP methods are shown in Figures 6(g-j), respectively. As in this analysis the image information of the metallic ring is an important factor, hence, difference images are also incorporated in order to clearly understand the strength and weakness of the comparing methods. The difference image is calculated by directly subtracting the corrected slice from the corrupted slice. The difference images thus obtained by using the MWPN, WF, RCHT and RCP methods are shown in Figures 6(k-n), respectively. The MWPN method is almost successful in eliminating the ring artifacts while maintaining the metallic ring structure. The difference image (marked by arrow and box in Figure 6(l)) produced by the WF method indicates that the regions near the two edges are distorted because of the use of longer length mother wavelets. It is interesting that this distortion can be clearly observed in the difference image domain rather than in the reconstructed image domain. On the other hand, due to the cartesian-polar and polar-cartesian conversions, the RCHT method (Figure 6(m)) losses some high frequency information. Though the RCP method also requires the conversion processes, it does not extract any high frequency image feature [22] as the polar-cartesian transformation takes place in the difference image domain. This distinct feature makes the RCP method better than the RCHT method as clearly demonstrated in Figures 6(j), (n).

### Small high contrast object located exactly at the iso-center

**Figure 7:**
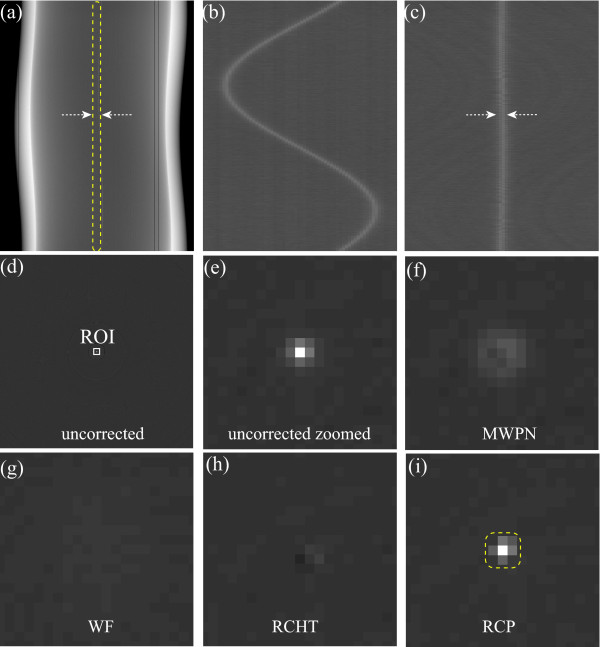
**Removal of ring artifacts from a uniform phantom image with a gold wire located at the iso-center**. (a) Uncorrected, initial projection image of a gold wire placed at near of the iso-center of a uniform phantom, (b) zoomed view of the ROI selected in (a). (c) Modified ROI. (d) The reconstructed uniform phantom image with a gold wire located at the iso-center, (e) magnified view of the ROI in (d). (f-i) Corrected ROIs by using the MWPN (*L_max _*= 1, *k*_0 _= 8.3, *T_HN _*= 0.20 and *N *= 4), WF (*L *= 4, wname='db41' and *σ *= 6.0), RCHT (*W *= 125 and *T *= 0.0019), and RCP methods, respectively. Same window settings 'C/W = 0.5/1.0' are used for (d-i).

Finally, the comparative analysis of the four methods is ended with the test on an uniform phantom with a gold wire (diameter: 20 *μ*m, FPD (C7942CA-02) pixel size: 50 *μ*m) inserted in it. It should be noted that the previous CT images (Figures 3, 5 and 6) are obtained from the first FPD (C7943CA-02). In fact, this experiment is performed to check the safety of the ring correction algorithms when a small high contrast object is located at the iso-center. It is extremely difficult to image such a small object perfectly placed at the iso-center. In our experiment we could image the gold-wire only close to the iso-center. As in this present analysis, the interest is to study the performance of all the comparing methods when a small high contrast object is located at the iso-center, therefore, some modifications are needed in the so obtained sinogram in order to achieve the aforesaid interest. The uncorrected sinogram is shown in Figure 7(a). Now, a region of interest is chosen around the center pixel of the sinogram and the magnified view of the ROI is displayed in Figure 7(b). It is illustrated from this zoomed ROI that the projection of the gold wire in the sinogram looks like a sine pattern. Now, some operations are performed only on the ROI of the sinogram to convert the sine wave pattern into a stripe pattern (Figure 7(c)) of 7-pixel width. This conversion ensures that the reconstructed image now contains the gold wire at the iso-center as shown in Figure 7(d). Next, a small ROI (21 pixels × 21 pixels) is chosen and the zoomed view is demonstrated in Figure 7(e). The corrected images are demonstrated in Figures 7(f-i) obtained by using the MWPN, WF, RCHT and RCP method, respectively. It is clearly visualized from Figures 7(f-i) that except the RCP methods, the other three methods unfortunately remove the gold-wire from the iso-center. As the gray values of the image elements around the center of rotation are above the upper threshold (*T_max_*), therefore, these image elements are not affected by the RCP correction method and therefore, it retains the gold wire. Therefore, the preservation of a lesion type structure at or very close to the iso-center can only be assured by the RCP method provided that the upper threshold (*T_max_*) is lower than the intensities of the lesion type structure.

### Comparison of ring artifact correction methods using objective indices

Here, two numerical indices are used to quantify the corrected image quality. One is the conventionally used peak signal-to-noise ratio (PSNR) and the other is the mean structural similarity (MSSIM) [27]. The first index PSNR is directly related to the intensity differences between the reference and corrected images. On the other hand, the latter is shown to be more correlated with the perceptual quality of an image. It considers luminance, contrast and structure similarity between the reference and corrected images to determine the value of the index. But evaluation of these two indices requires reference images, i.e., images free from ring and radiant artifacts. In CT imaging reference image is hardly available and, therefore, in this work one synthetic (computer simulated head phantom) sinogram image is used. In addition, two stripe-corrected versions of real FPD-CT sinogram images (rat abdomen and bone) are selected for comparison. These two images are obtained from a new and nearly error free FPD, so that the stripes present in the sinograms can be easily corrected by the state-of-the-art algorithm. Then, different types of stripe structures including single, band, stripes from defective and mis-calibrated detector elements are superimposed on the reference sinogram images to generate corrupted sinogram images. Note that the stripe structures added to the different reference images are also characteristically different. The comparing sinogram-processing methods are applied on these corrupted sinogram images and the post-processing methods, on the other hand, are applied on the CT images reconstructed from the corrupted sinograms. Now the above mentioned two indices can be calculated to quantify the visibility of errors between the reference and corrected CT images.

The first quantitative index PSNR is defined as

$$\text{PSNR}=20\log_{10}\left(\frac{M_{I}}{\sqrt{(}MSE)}\right)\;\text{dB}$$

where,

$$MSE\;=\;\frac{1}{PQ}\overset{P-1}{\underset{i=0}{\sum }}\overset{Q-1}{\underset{j=0}{\sum }}{\left[X\left(i,\;j\right)-Y\left(i,\;j\right)\right]}^{2}$$

and *X*|_*P *× *Q *_and *Y*|_*P *× *Q *_are the reference and corrected CT images, respectively and, *M_I _*is the dynamic range of the reference image.

On the other hand, to calculate the MSSIM index at first the reference and corrected CT images are windowed and two signals, i.e., **x **(**x **= [*x*_1 _*x*_2_......*x_N_*]) and **y **(**y **= [*y*_1 _*y*_2_......*y_N_*]) are generated. Then these two signals are weighted using a Gaussian weighting function, **w **= [*w*_1 _*w*_2_......*w_N_*] with a standard deviation of 1.5 samples, where $\sum_{i=1}^{N}w_{i}=1$. Then the estimates of local statistics of **x **and **y **are calculated as: $\mu_{x}=\frac{1}{N}\overset{N}{\underset{i=1}{\sum }}w_{i}x_{i}$, $\mu_{y}=\frac{1}{N}\overset{N}{\underset{i=1}{\sum }}w_{i}y_{i}$,

$$\begin{array}{c} \sigma_{x}=\sqrt{\frac{1}{N-1}\overset{N}{\underset{i=1}{\sum }}w_{i}{\left(x_{i}-\mu_{x}\right)}^{2}},\\ \sigma_{y}=\sqrt{\frac{1}{N-1}\overset{N}{\underset{i=1}{\sum }}w_{i}{\left(y_{i}-\mu_{y}\right)}^{2}},\\ \sigma_{xy}=\frac{1}{N-1}\overset{N}{\underset{i=1}{\sum }}w_{i}\left(x_{i}-\mu_{x}\right)\left(y_{i}-\mu_{y}\right). \end{array}$$

The SSIM index between signals **x **(**x **= [*x*_1 _*x*_2_......*x_N_*]) and **y **(**y **= [*y*_1 _*y*_2_......*y_N_*]) is calculated as [27]

$$\text{SSIM}\;=\;\frac{\left(2\mu_{x}\mu_{y}+C_{1}\right)\left(2\sigma_{xy}+C_{2}\right)}{\left(\mu_{x}^{2}+\mu_{y}^{2}+C_{1}\right)\left(\sigma_{x}^{2}+\sigma_{y}^{2}+C_{2}\right)}$$

where, *C*_1 _= (*K*_1_*M_I_*)^2^, *C*_2 _= (*K*_2_*M_I_*)^2^, *K*_1 _= 0.01 and *K*_2 _= 0.03. Finally, the mean SSIM (MSSIM) is evaluated as

$$\text{MSSIM}\;=\;\frac{1}{M}\overset{M}{\underset{j=1}{\sum }}\text{SSIM}\left(x_{j},\;y_{j}\right)$$

where, **x***_j _*and **y***_j _*are the image contents at the *j*-th local window; and *M *is the number of local windows in the image. Now, we calculate the PSNR and MSSIM for the three reference and corrected CT images, and the calculated values are shown in Table 1. The calculated values in this table indicate that the values of the PSNR and MSSIM for the MWPN method are quite satisfactory, but impressive results are not obtained for the other three (e.g., WF, RCHT and RCP) methods. But if we add the proposed pre-correction technique to correct the varying intensity ring structures, then the PSNR and MSSIM significantly improve for these three methods. These improvements observed in the PSNR and MSSIM indices are due to the addition of the pre-correction technique which is a part of our SBRC method.

**Table 1:** Quantitative performance of the comparing algorithms

	Head phantom		Rat abdomen		Bone (figure 4(b))	
**Methods**	**PSNR**	**MSSIM**	**PSNR**	**MSSIM**	**PSNR**	**MSSIM**

MWPN	39.04	0.97	38.16	0.96	38.73	0.93

WF	32.35	0.81	35.83	0.93	30.95	0.72

RCHT	27.96	0.82	31.71	0.83	29.79	0.71

RCP	25.48	0.77	28.91	0.78	31.05	0.73

WF with proposed pre-correction	37.39	0.94	37.40	0.95	38.25	0.93

RCHT with proposed pre-correction	38.15	0.96	40.54	0.97	39.78	0.95

RCP with proposed pre-correction	39.41	0.97	37.88	0.95	40.84	0.96

Proposed SBRC	40.67	0.98	42.03	0.99	40.95	0.96

### Discussion

Although good results were demonstrated by the authors using the WF, RCHT and RCP methods [2,6,22,23], these algorithms, however, when tested using our original, uncorrected CT images (e.g., Figures 3 and 5) the results found were not encouraging. Also the numerical results shown in Table 1 indicate the incapability of the WF, RCHT and RCP methods in properly correcting the ring artifacts. This poor performance may happen due to the presence of strong varying intensity rings in our CT images generated from the large area CMOS FPD and low X-ray exposure levels [18]. Thus, some modifications may be needed in the WF, RCHT and RCP algorithms in order to suit these algorithms for such CT images. If the varying intensity rings can be removed anyway, then the corrected images by utilizing these three methods assure good diagnostic quality as demonstrated in Figure 6 and Table 1.

In this paper, the performance of different correction methods are investigated by applying different conditions/situations, e.g., presence and absence of varying intensity rings, rings/band rings at the center of rotation, rings/band rings in micro- and dental-CT images, rings far from the center of rotation, rings located near to a highly structural object and edges of two different contrast medium, presence of a high contrast small object at the iso-center, effect of unique parameter setting on different slices of a 3-D CBVCT image, and effectiveness of the correction algorithms using different FPDs etc. Also different kinds of objects, e.g., electrolytic capacitor, rat abdomen, bone are used in this study to check the effectiveness of the comparing methods in various samples. Two numerical indices are used to measure the ability of these methods in correcting the ring structures. Some characteristics of the ring removal methods are summarized in Table 2. From this extensive analysis it can be stated that though the MWPN method cannot remove the band rings (e.g., in Figure 4(g)) effectively, the overall performance of the modified wavelet plus normalization (MWPN) method is better than that of the other three techniques. Modified wavelet method without normalization can eliminate only the strong ring artifacts, whereas the WF method is not very appropriate for the removal of varying intensity rings. Also, this method distorts the image at the center of the image. The RCHT and RCP methods also suffer from the varying intensity rings. If a pre-correction of the varying intensity rings is performed, it is then possible to obtain acceptable performance from the WF, RCHT and RCP methods, especially from the RCP method. The RCP method is better than the WF and RCHT methods in some perspectives, e.g., in the removal of band ring structures, retaining high contrast structure at the iso-center. It also prevents the information losses in the coordinate transformation unlike the RCHT method. It is also noticed that all the methods can be applied on dental-CT images. Though the corrected images obtained from the dental-CT are not completely ring-free for some of the methods, it does not indicate the weakness of these algorithms in dental-CT application. The failure of these methods in suppressing the ring artifacts from the dental-CT images is due to the presence of band rings. Without any doubt, presence of a small object at the iso-center is a special issue and may rarely be observed in a real condition. The RCP method, however, is found successful in this particular case. In the comparative analysis, we varied the parameters in order to obtain the optimal results. It is observed from the searching of optimal parameters that for the MWPN method the corrected images are highly sensitive to the parameters' setting, in particular to the value of the span factor *N *used in smoothing the sum curve. On the other hand, the rest three methods are less sensitive to the parameters' setting.

**Table 2:** Performance summary of the comparing algorithms in some aspects (Yes = '√' and No = '×')

Performance index	MWPN	WF	RCHT	RCP	SBRC
Is able to remove sharp varying intensity rings?	√	×	×	×	√

Is able to remove weak mis-calibrated rings?	√	√	√	√	√

Is able to remove radiant artifacts?	√	√	×	×	√

Is the corrected CT image free from blurring?	√	×	×	×	√

Is able to remove band ring artifacts?	×	×	×	√	√

Is diff. image is free from object information?	√	×	×	√	√

Is able to keep high contrast structure at iso-center?	×	×	×	√	×

Is applicable in dental-CT?	√	√	√	√	√

Is less sensitive to parameters setting?	×	√	√	√	√

Is able to suppress artifacts generally from all slices?	×	×	×	×	×

It is clear from Table 2 that none of the methods is suitable for correcting all types of the ring artifacts. A combination of two methods (e.g., MWPN and RCP) can be used to achieve the diagnostic quality of the corrected CT images. This type of combined (both sinogram- and post-processing algorithms) correction schemes are often installed in commercial CT scanners to obtain satisfactory results. But in one point all the methods are same, i.e., all these four methods cannot completely eliminate the ring artifacts from all slices of a 3-D CBVCT image. It is observed that some 2-D slices (e.g., a CT image in Figure 5(a)) in a CBVCT image may be severely corrupted and these algorithms particularly suited to multi-slice CT or processing CBVCT images slice by slice are in-appropriate to clean such slices.

**Figure 8:**
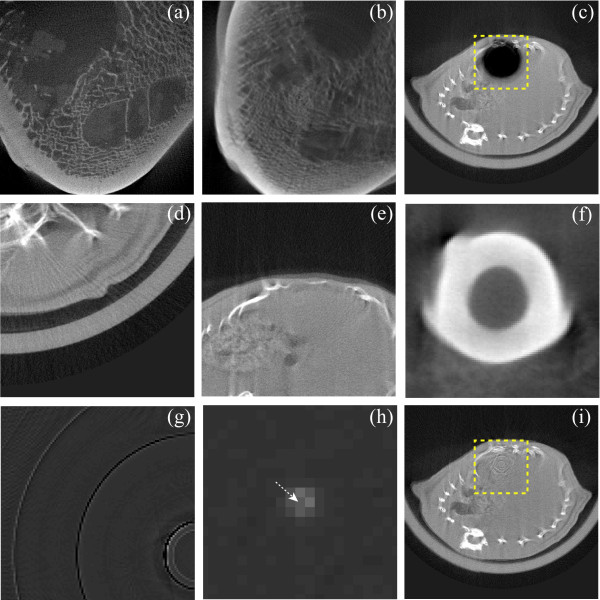
**Removal of ring artifacts from the analyzed CT images by applying the SBRC method and effect of using wavelet-analysis-based method on cone beam CT volume images**. (a) Corrected bone image obtained from micro-CT machine (*r_max _*= 15, *r_min _*= 1.5 and *l_m _*= 5) (C/W = 0.4132/0.2994), (b) corrected bone image from dental-CT machine (*r_max _*= 15, *r_min _*= 1.5 and *l_m _*= 5) (C/W = 0.4714/0.1998), (c-e) corrected three rat abdomen slices (*r_max _*= 15, *r_min _*= 1.5 and *l_m _*= 5) (C/W = 0.2628/0.0481), (f) corrected ROI of the rabbit image (*r_max _*= 15, *r_min _*= 1.5 and *l_m _*= 5) (C/W = 0.6716/0.5827), (g) difference image between the uncorrected and corrected ROI of rabbit (C/W = 0.6716/0.5827), (h) corrected uniform phantom image with a gold wire located at the iso-center (*r_max _*= 15, *r_min _*= 1.5 and *l_m _*= 5) (C/W = 0.5/1.0). (i) Corrected first rat abdomen slice using the wavelet-analysis-based method [13] (C/W = 0.2628/0.0481).

From the above discussion, it is clear that considering different types of ring artifacts separately can be a solution to eliminate some lackings of the ring correction algorithms. Now, our classification based SBRC method is applied to correct the CT images presented above and the results obtained are presented in Figures 8(a-h). Also the PSNR and MSSIM values for the SBRC method are shown in Table 1. It is observed from Figures 8(a-h) that this method can successfully remove the ring artifacts from the CT images under consideration. The PSNR and MSSIM indices also indicate the superiority of the SBRC method over the other four comparing methods. The SBRC method principally uses three parameters, *r_max_*, *r_min_*, and *l_m_*, to generate a ring-free image. Amongst these parameters, *r_max _*and *r_min _*denote the boundary conditions. In this paper, *r_max _*= 15, *r_min _*= 1.5 and *l_m _*= 5 are chosen for all the CT images. The unique setting of these three parameters and the corrected image quality indicate that the corrected images by the SBRC method are less sensitive to the parameters setting. But two drawbacks are still noticed. First, the intensity of the gold wire at the iso-center is decreased as shown in Figure 8(h). Such lower intensity of an object may give a false decision about the properties of the object, e.g., a gold wire can appear as a low contrast object in the corrected CT image. Second, like other methods discussed above it cannot erase the ring artifacts from the severely corrupted tomographic slice as shown in Figure 8(c). As stated before, this type of algorithm is particularly suited to the multi-slice geometry and uses only 1-D pixel information to correct the ring artifacts and as a result, cannot correct the 3-D CBVCT images completely. To achieve the required quality for all the slices, the information from the 2-D neighboring pixels must be exploited by an algorithm. Figure 8(i) shows the corrected rat abdomen slice (uncorrected slice is shown in Figure 5(a)) using such an algorithm proposed in [13]. This method performs 2-D wavelet analysis on the flat-field image [13] to detect the defective pixels in a 2-D FPD and uses correlation between both horizontal and vertical neighboring pixels to correct the responses. As can be seen, this this method evidently removes the severe ring artifacts and retrieves the obscured image information (as marked by box in Figure 8(i)).

The overall performance of a ring artifact removal technique can be evaluated by its effectiveness and the computation time it requires. The computation time will be an important factor for real time processing. A comparison of these four methods in terms of computation time reveals that the MWPN method requires the shortest time to generate a corrected images. The RCP method is faster than the WF method. On the other hand, excessive computational complexity in the RCHT method makes it the slowest amongst these four methods.

## Conclusion

This paper has dealt with the performance comparison of different ring artifact correction algorithms (with FPD based CT images) selected from the two categories of reported techniques, namely, sinogram domain processing and post-processing. Real CT images from multiple FPDs have been used to test their effectiveness. As the ring artifacts appear in diverse forms, e.g., varying intensity and mis-calibration rings, band artifact, radiant artifact, rings in highly structural object, rings in between different contrast medium, therefore, none of the algorithms were found completely satisfactory for suppressing the ring artifact as clearly evident from Table 2. However, the overall performance of the MWPN method is better than the WF, RCHT and RCP methods as is evident from Table 1. This MWPN method is actually an improved version of the modified wavelet method. Incorporating the normalization technique with the proposed modified wavelet method effectively improves the image quality. The wavelet-Fourier method is particularly weak in suppressing the strong varying intensity ring artifacts and, on the other hand, the RCHT and RCP methods can reduce the strength of the varying intensity ring artifacts but fail to eliminate them completely. Therefore, low PSNR and MSSIM values are obtained for the WF, RCHT and RCP methods. If varying intensity rings are pre-corrected, then the performance of these methods, especially that of the RCP method becomes quite satisfactory as can be observed from Table 1. Band ring removal is still a critical issue in ring artifact research, and it is observed from our extensive analysis that only the RCP and a newly introduced SBRC method [17] can successfully remove this band ring artifact. It is also remarked that considering two different types of ring artifacts (e.g, varying intensity and mis-calibration) separately and adopting appropriate correction schemes for each type is one way to improve the accuracy of these correction methods. Moreover, it is noticed that a ring removal algorithm using neighborhood pixel information in 2-D space, e.g., [13] is required to eliminate effectively the ring artifacts from all the slices of a 3-D CBVCT image.

## Competing interests

The authors declare that they have no competing interests.

## Authors' contributions

EM carried out the implementation of the idea, contributed in the analysis of the comparative study, and drafted the manuscript. JK participated in the acquisition of FPD-CT data and helped in accurate reconstruction of some images presented in the manuscript. SY participated in the design of the study and interpretation of data, and was involved in critically revising the manuscript. MK conceived of the study, and participated in its design, analysis and interpretation of data, and helped to draft and finalize the manuscript. All authors read and approved the final manuscript.
